# Total Water Intake from Beverages and Foods Is Associated with Energy Intake and Eating Behaviors in Korean Adults

**DOI:** 10.3390/nu8100617

**Published:** 2016-10-04

**Authors:** Kyung Won Lee, Dayeon Shin, Won O. Song

**Affiliations:** 1 Department of Food Science and Human Nutrition, Michigan State University, 469 Wilson Road, Trout FSHN Building, East Lansing, MI 48824, USA; kyungwon@msu.edu; 2 Department of Nutrition & Dietetics, University of North Dakota, 221 Centennial Dr, Stop 8237, Grand Forks, ND 58202-8237, USA; dayeon.shin@und.edu

**Keywords:** water intake, beverage consumption, energy intake, eating behavior, Korean adults

## Abstract

Water is essential for the proper functioning of the body. Even though a recommendation exists for adequate water intake for Koreans, studies identifying actual water intake from all beverages and foods consumed daily in the Korean population are limited. Thus, we estimated total water intake from both beverages and foods and its association with energy intake and eating behaviors in Korean adults. We used a nationally representative sample of 25,122 Korean adults aged ≥19 years, from the Korean National Health and Nutrition Examination Survey 2008–2012. We performed multiple regression analyses, adjusting for sociodemographic and health-related variables to investigate the contribution of overall energy and dietary intakes and eating behaviors to total water intake. The mean total water intake excluding plain water was 1071 g (398 g from beverages and 673 g from foods) and the estimated plain water intake was 1.3 L. Among Korean adults, 82% consumed beverages (excluding plain water) and these beverages contributed to 10% of daily energy intake and 32% of total water intake from beverages and foods. For every 100 kcal/day in energy intake, water intake consumed through beverages and foods increased by 18 g and 31 g, respectively. Water intake from beverages and foods was positively associated with energy from fat and dietary calcium, but inversely associated with energy density and energy from carbohydrates. When there was a 5% increase in energy intake from snacks and eating outside the home, there was an increase in water intake from beverages of 13 g and 2 g, respectively. Increased daily energy intake, the number of eating episodes, and energy intake from snacks and eating outside the home predicted higher water intake from beverages and foods. Our results provide evidence suggesting that various factors, including sociodemographic status, dietary intakes, and eating behaviors, could be important contributors to the water intake of Korean adults. Findings from this cross-sectional analysis may provide insight into strategies for promoting adequate water intake among Koreans.

## 1. Introduction

Water is a crucial nutrient comprising 60% of human body weight [1,2]. It is well documented that water plays an essential role in the heathy functioning of the body, including regulation of body temperature, transport, digestion and absorption of nutrients, and excretion of wastes [3,4]. Water imbalance in the body is associated with adverse health consequences from mild thirst to severe dehydration, delirium, and even death [5]. Plain water intake is the major contributor to total water intake, though the ratios may vary among countries. Water may also be consumed from daily consumption of foods and beverages [2,6].

Many longitudinal and cross-sectional studies have been conducted on water intake in relation to dietary intakes, weight status, and disease. It has been found that increasing one’s intake of non-caloric beverages such as plain water and coffee, including the replacement of sugar-sweetened beverages (SSBs) with plain water, is inversely associated with weight gain [7,8,9] and the risk of type 2 diabetes [10]. A few investigations have suggested inverse associations of the amount of water intake with the risk of chronic kidney disease [11] and negative mood [12]. Additionally, some studies have indicated that water intake from foods and beverages is significantly associated with micronutrient (e.g., vitamins and carotenoids) as well as macronutrient (e.g., carbohydrate, protein, and fat) intakes [2,13,14]. However, most of these prior studies have been conducted in Western countries.

Acknowledging the importance of water intake in maintaining health and preventing disease, many countries suggest recommendations for water intake to encourage the public to drink adequate amounts of water. In the United States (US), the Adequate Intake (AI) of total water was established by adding estimated water intake from foods and beverages obtained from nationally representative data [15]. The European Food Safety Authority recommends an adequate intake of water based on the consideration of the observed national estimates of water intake and the desired urine osmolality [16]. In Korea, recently published the 2015 Dietary Reference Intakes (DRI) for Koreans offer an AI of water for the Korean population. The AI for Korean adults over 19 years of age ranges from 2.1 to 2.6 L/day and 1.8–2.1 L/day for men and women, respectively, with some variations among age groups. The AI of total water for Koreans was estimated by combining water intake from commonly consumed foods and fluid intake, which includes the median value of plain water and beverage intakes (excluding milk and other dairy products) and 200 mL of milk and other dairy products. However, actual water intake from milk and other dairy products as well as water intake from soup, which has a high water content, was not reflected in the calculation of the AI for total water [6].

The beverage industry in Korea has grown and is now a global trend. According to recent statistics on the most frequently consumed foods in Korea, carbonated beverages are ranked third, blended beverages fourth, and fruit/vegetable beverages eighth [17]. Moreover, there is an increasing tendency towards sales of bottled water, coffees, carbonated beverages, and tea-based beverages in Korea [18,19]. Although the importance of beverages including plain water has increased in the Korean diet, there is a limited body of knowledge regarding total water intake and water intake from beverages and foods in Korea. There have been some investigations on water intake and beverage consumption for Koreans, but these were limited to certain regions, small sample sizes [20,21], and specific beverages such as SSBs and coffee [22,23]. Thus, there is no representative picture of total water intake among the Korean population. Moreover, no studies have investigated water intake from the daily consumption of all foods and beverages among Koreans. Basic epidemiological research on estimated water intake from commonly consumed foods and beverages is needed to provide better guidelines for water intake in upcoming DRIs for Koreans.

Given the limited number of studies attempting to quantify total water intake among Korean adults, investigations on the associations of water intake from foods and beverages with dietary intake and meal consumption attributes might help to lay the foundation for future work establishing better recommendations for water intake in Koreans. Therefore, the purpose of this study is (1) to describe water intake from beverages and foods in respect to beverage consumption; (2) to identify certain sociodemographic and health-related characteristics that can predict water intake; and (3) to investigate water intake in relation to energy intake and eating behaviors using a nationally representative sample of Korean adults from the Korea National Health and Nutrition Examination Survey (KNHANES) 2008–2012 data.

## 2. Methods

### 2.1. Data Source and Study Sample

The KNHANES conducted by the Korea Centers for Disease Control and Prevention (KCDC) and the Korean Ministry of Health and Welfare provide ongoing surveillance. The KNHANES data were collected to monitor the health and nutritional status among Koreans and to provide meaningful baseline data for health and nutrition policy design in Korea [24,25]. To establish nationally representative samples of the civilian noninstitutionalized population of Korea, the sampling plan of the KNHANES is based on a multi-stage clustered probability design [26].

Data from the KNHANES 2008–2012 were used in this cross-sectional study to identify important contributors of water intake in Korea. All participants who were older than 19 years of age with a nutrition survey including a single 24-h dietary recall and health interview were eligible for inclusion in this study (*n* = 30,642). We excluded those with <500 or >5000 kcal/day of total energy intake (*n* = 469) and pregnant and lactating women (*n* = 612). We also excluded those who had incomplete information on socio-demographic characteristics (*n* = 2816), health-related behaviors (*n* = 1370), and reported implausible total water intake besides plain water intake (>3466 mL) (*n* = 253), for a final analytic sample of 25,122 (10,184 men and 14,938 women).

The KNHANES used in this study was approved by the KCDC Institutional Review Board. All subjects gave their informed consent and participated voluntarily.

### 2.2. Water Intake Assessment

Four variables reflecting water intake were used in the present study: (1) water intake from beverages excluding plain water; (2) water intake from foods; (3) total water intake from beverages and foods other than plain water; and (4) usual plain water intake. As part of the 24-h dietary recall data, the water content of all beverages and foods consumed by participants in the KNHANES were publicly released [27]. Estimated water intake from beverages were calculated based on the total content of water in all types of fluids such as milk, unsweetened coffee and tea, 100% fruit/vegetable juice, SSBs, alcoholic beverages, and diet beverages. Information on usual plain water intake was collected by asking participants using the food-frequency questionnaire (FFQ)-type question, “How much plain water do you usually drink a day?” after 24-h dietary recalls.

Based on the KNHANES coding scheme [24] and previous classifications of beverages [28,29,30,31], we grouped all beverages into six categories (milk, unsweetened coffees/teas, 100% fruit juices, SSBs, alcoholic beverages, and diet beverages). The SSBs category was further divided into five subgroups (sodas, fruit drinks, sweetened coffees/teas, sports/energy drinks, other SSBs). To understand beverage consumption in the Korean adult population, we calculated four estimates (per capita and per consumer): average intake of beverages (mL), average water intake from beverages (g), and the contribution of beverages to daily total energy (%), and total water intake excluding plain water (%).

### 2.3. Dietary Intake and Eating Behaviors

To describe how each water variable varied according to dietary intakes and eating behaviors, we used several variables that may reflect energy intakes, dietary constituents, and meal consumption behaviors. We estimated total energy intake (kcal/day), % energy from macronutrients (carbohydrate, protein, and fat), dietary fiber, calcium, and sodium consumed [32]. We also used energy density (kcal/g) for all beverages (not including plain water) and foods [2], and estimated the amounts of beverages and all non-beverage foods consumed.

We used several eating behavioral variables based on previous literature [33,34] to assess meal consumption attributes of study subjects in relation to water intake. They were described by the frequency of eating episodes and % energy from main meals and snacks. The KNHANES data provided information on eating episodes reported by survey participants. We identified each eating episode named by respondents with breakfast, lunch, and dinner considered as the main meals. All other non-main meal eating episodes were considered as snacks. Eating episodes reported at different clock times were classified as a single eating episode regardless of the number or quantity of foods or beverages consumed. Based on this information, we estimated the number of total eating episodes, snack and beverage-only episodes, and % energy from main meals and snacks. Length of ingestion period was calculated from the first eating event to the last eating episode reported. Additionally, eating-out behaviors were measured using the information on locations where food and beverages were consumed as collected in the KNHANES. We classified all beverages and foods consumed into two categories based on the eating location and calculated 24-h energy intake at home and outside the home, respectively.

### 2.4. Potential Covariates

To determine an independent association between all water variables and dietary intakes and eating behavioral factors, potential confounders were included in our statistical model. Socio-demographic variables included: age (19–29, 30–49, 50–64, ≥65 years of age); income (income was calculated according to total household income of residents and then divided into quartiles from poorest to wealthiest; low, mid-low, mid-high, or high) [24]; education level (elementary school graduates, middle school graduates, high school graduates, or more than college graduate). Diet and health-related behavioral variables included: smoking status (never smoked, former smoker, or current smoker), the day of recalled intake (Monday-Thursday or Friday-Sunday), regular physical activity (yes or no; having physical activity was defined as walking ≥5 times a week for ≥30 min each time), and body mass index (BMI; kg/m^2^) (calculated as weight in kilograms divided by square of height in meters; ≤normal weight, <23; overweight, 23 to <25; or obese, ≥25) [35].

### 2.5. Statistical Analyses

We used SAS (version 9.4, SAS Institute Inc., Cary, NC, USA) for statistical analyses in this study. All statistical tests were two-tailed and *p* < 0.05 were considered statistically significant. We used SURVEY procedure with sample weight, stratum, and primary sampling unit recommended by the KNHANES analytical guidelines to account for unequal selection probabilities resulting from complex survey design and non-response [24,26].

We computed the adjusted means using multiple linear regressions to compare all types of water intake according to sociodemographic and health-related characteristics (Table 1). Frequency (weighted percentage) and means (standard errors) were calculated to depict beverage consumption in Korean adults with regard to the prevalence of consuming different types of beverages and water intake, % total water intake excluding plain water, and % total daily energy from each beverage category (Table 2). Multiple logistic regressions were conducted to determine the associations of sociodemographic and health-related variables with odds of various sorts of beverages. We calculated multivariable-adjusted odds ratios with 95% confidence intervals after controlling for covariates such as sex, age, income, education level, smoking status, the day of recalled intake, regular physical activity (categorical), and total energy intake (continuous) (Table S1). We also conducted multiple linear regression analyses to explore the independent association of energy and dietary intake and eating behaviors with all components of water intake (Table 3 and Table 4). For these analyses, important contributors to water intake mentioned above were included in our regression models as covariates.

## 3. Results

The daily amount of total water intake from beverages and foods was 1071 g (1.1 L) and the mean usual plain water intake was 1.3 L among Korean adults (Table 1). Usual plain water intake reported did not vary depending on sociodemographic characteristics and health-related behaviors, except for smoking status. Current smokers usually consumed more plain water than non-smokers and former smokers (*p* < 0.05). Compared to women, men consumed a higher amount of water from beverages and foods. Age was negatively correlated to water from beverage and total water intake, but positively correlated to water consumed through foods (*p* < 0.01). Income and education levels were positively associated with all water variables (*p* < 0.01). Like usual plain water intake, current smokers consumed higher amounts of water from beverages and total water, but lower amounts of water from foods than non-smokers (*p* < 0.01). Water intake from beverage and total water intake excluding plain water were higher on weekends than weekdays (*p* < 0.01). Those undertaking regular physical activity consumed more water from foods and total water compared with their counterparts (*p* < 0.01), but there was no association with water intake from beverages and usual plain water intake.

Korean adults consumed 451.0 ± 4.5 mL of beverages and 395.8 ± 4.0 g of water from beverages per capita each day (Table 2). Beverages other than plain water contributed to about 10% of total energy intakes and 32% of total daily water intake besides plain water. The prevalence of consuming beverages was about 82%. Approximately, 49% of people reported consuming SSBs, more than 32% reported consuming unsweetened coffee/tea, and 26% people reported consumption of milk. The largest per capita beverage consumption were SSBs (139.7 ± 2.2 mL), alcoholic beverages (116.4 ± 2.9 mL), and unsweetened coffee/tea (108.0 ± 2.2 mL). In addition, SSBs (122.5 ± 1.9 g) accounted for the greatest per capita water intake from beverages, followed by unsweetened coffee/tea (100.2 ± 2.1 g), and alcohol beverages (99.2 ± 2.5 g). However, per consumer, both the amount of water from beverages and the percentage of energy consumed as alcoholic beverages were the greatest contributor to water and energy intake from beverages. There was a significant contribution of water intake by unsweetened coffee/tea, although its contribution to total daily energy intakes stayed <1% for both per capita and per consumer. Since diet beverages were rarely consumed by all individuals, meaningful numbers related to this beverage category could not be determined.

The prevalence of consuming different types of beverages according to sociodemographic and health-related characteristics was presented elsewhere (see Table S1). Women were more likely to consume milk, unsweetened coffee/tea, and SSBs (*p* < 0.01), but less likely to have alcohol compared to men (*p* < 0.01). Compared to younger adults (19–29 years of age), the consumption of unsweetened coffee/tea and alcohol was higher in other age groups, however, there was lower milk and 100% fruit juice consumption in adults aged over 30 years compared with younger adults (*p* < 0.01). The prevalence of consuming milk and unsweetened coffee/tea increased with level of income and education. Those with a higher education were more likely to consume milk, unsweetened coffee/tea, and even SSBs (*p* < 0.05). Current smokers and individuals reporting no physical activity were less likely to consume milk but more likely to consume SSBs.

Total daily energy intake was positively correlated to water intake from beverages and foods and total water intake excluding plain water (*p* < 0.01) (Table 3). The 17.8 ± 0.5 g and 30.7 ± 0.5 g increases in water intake from beverages and foods were observed for each 100 kcal increment in energy intake. Energy density (kcal/g) of all beverages and foods were negatively correlated to total water content of beverages and foods (*p* < 0.01). Percent energy as protein was positively related to water intake from foods (*p* < 0.01), but negatively related to water from beverage and total water intake (*p* < 0.01). While percent energy as fat was positively correlated with all water variables (*p* < 0.01), percent energy as carbohydrates was negatively correlated (*p* < 0.01). Dietary fiber and sodium were significantly associated with higher water intake from foods and total water intake (*p* < 0.01), but they showed negative associations with water intake from beverages. Intake of usual plain water had no significant association with water intake from beverages and foods. However, water from foods and beverages were significantly related to each other and to total water intake excluding plain water after adjusting for covariates. All water intake from different types of beverages were positively associated with intake of water from beverages (*p* < 0.01), but such associations were observed in water from foods. The greatest contributors to water intake from beverages were water consumed as alcoholic beverages and 100% fruit juices. When there was a 100 g/day increase in the consumption of alcohols and 100% fruit juices, there was an increase in water consumed through beverages of 90.9 ± 0.8 g and 87.5 ± 4.4 g, respectively.

Independent associations of eating behaviors with contribution to water intake are listed in Table 4. The number of unique beverages consumed in the 24-h dietary recall was positively associated with water intake from beverages (*p* < 0.01), but negatively associated with water from foods (*p* < 0.01). In case of the number of non-beverage foods consumed, the reverse association with water consumed from foods was found (*p* < 0.01). Particularly, the number of different types of beverages was the most important contributor of higher intake of water from beverages. When the number of unique beverages consumed increased, water consumed from beverages increased by 211.2 ± 2.7 g. The number of beverage-only episodes and eating duration were positively related to water consumed from beverages and total water intake besides plain water (*p* < 0.01), but negatively related to water content consumed through foods (*p* < 0.01). Reported total daily energy from main meals was negatively correlated with water intake from beverages (*p* < 0.01), but positively correlated with water consumed from foods and total water (*p* < 0.01). The number of total eating episodes and snack episodes were positively related to all water intakes (*p* < 0.01). A similar trend was observed for 24-h energy intake from snacks and reporting a snack in the 24-h dietary recall. Increasing the energy consumed outside the home had positive associations with intakes of water from beverages and foods and total water intake other than plain water (*p* < 0.01). For each 5% increase in energy from snacks and eating outside the home, the 30.2 ± 1.2 g and 12.6 ± 0.6 g, respectively, increases in water intake from beverages were found.

## 4. Discussion

This study described total water intake from beverages and foods consumed daily and explored the significant associations of water intake with energy and dietary intakes as well as eating behaviors using 25,122 weighed dietary information from Korean adult subjects in a nationally representative sample of the KNHANES 2008–2012. These findings might help to shed light on the important contributors to water intake and aid in understanding the current status of total water intake among Korean adults. 

On average, Korean adults consumed 1.1 L of total water, excluding plain water (0.4 L of water from beverages and 0.7 L of water from foods, respectively), based on the 24-h dietary recall data. Adding 1.3 L of usual plain water intake estimated from the FFQ-type question, the average amount of total water consumed daily by Koreans was 2.4 L, satisfying the AI of water intake for Koreans. This is somewhat lower than 3.1–3.2 L, the total water intake of Americans estimated from the National Health and Nutrition Examination Survey (NHANES) 2005–2006 data [2,13]. In terms of water intake including all beverages and plain water, the average was 1.7 L, similar to that of China (1.7 L) and Japan (1.5 L), whereas it was slightly lower than that of Western countries, which range from 1.9 L in Spain to 2.5 L in Germany [36]. However, it is difficult to combine plain water intake as estimated using the FFQ-type question with water intake collected from the 24-h dietary recalls, since information on the plain water intake of Koreans was collected by asking an FFQ-type question “How much water do you usually drink a day?” right after the 24-h dietary recall. In addition, Sebastian et al. reported that FFQ-type questions have limitations in that they tend to over-report dietary and energy intakes and even water intake compared to the 24-h dietary recall. Thus, they proposed that 24-h dietary recalls were more accurate for measuring water intake; therefore, continual development and improvement of the methodology are required [37].

We found that those who were younger, had higher income and education, and exercised regularly showed higher intakes of total water excluding plain water. These sociodemographic and health-related characteristics were major determinants of not only water intake but also better dietary quality among Koreans [38]. Higher water intake from beverages and usual plain water intake in current smokers were comparable to the results of previous studies [2,39,40]. There was no difference in water from beverages associated with regular physical activity, but water from foods and total water intake besides plain water were significantly higher among those who engaged in sufficient physical activity. It can be assumed that such a difference in water intake from foods results from differences in fruit and vegetable consumption, which are well-known sources of water in foods, between those who reported no physical activity and regular physical activity. Some Korean studies have reported that physically active people put more effort into reading nutrition labels and taking vitamin and mineral supplements [41]. In addition, vegetable and seafood patterns were more likely to be associated with physical activity [42]. We might conclude from previous literature that those who exercise regularly might follow a healthier diet compared with those who are not physically active.

It has been well-documented that vegetables and fruits are high-water-content foods, while oils, butter, and sugars contain the least water [43]. In other words, less high-fat intake or greater fruit and vegetable intake might be related to higher intake of water from food. Additionally, dietary energy density is inversely associated with higher consumption of fruits and vegetables [44]. This was supported by our result indicating a significantly negative association of water from foods with the energy density of all non-beverage foods. Water content of fruits and vegetables accounted for most of the water intake from foods in the Korean diet. Considering that the consumption of fruits and vegetables has a significantly positive association with water consumed from foods, adequate intake of water through foods can be achieved by higher consumption of fruit and vegetables. In our study, an increase in dietary sodium intake had a positive association with all components of water intake. Our finding was supported by the fact that an increased concentration of plasma sodium resulting from intakes of dietary salt increases fluid intake by stimulating thirst to maintain fluid homeostasis in our body [45]. In contrast, however, Kant et al. reported that dietary sodium intake was positively associated with water from foods but negatively associated with water from beverages and total water intake excluding plain water [2]. Different study populations used in both studies gave quite different results. Based on our findings, total daily energy intake from beverages in Korean adults was about 10%, which was only half of the result for Americans, who consume 20% of their daily energy from beverages [46]. The extent to which these differences in food consumption behaviors, particularly beverage-related, may cause the opposite associations between dietary sodium and intakes of total water and water consumed from beverages.

Another interesting result was observed in relation to eating behaviors and water intake. Reported snacking at the time of recall and 24-h energy from snacks were positively correlated with water intake from beverage. It is well-known that higher consumption of beverages has a significant association with increased frequency of snacking [47]. In a previous study on Korean dietary patterns, it was reported that most beverages, excluding alcoholic beverages with a meal and coffee immediately after a meal, could not be included as a part of main Korean dietary patterns. This indicated that most beverages were typically ingested not as a part of main meal but as a snack [48]. Similarly to snacking, eating outside the home was positively correlated with water consumed from beverages. This could be supported by the previous study indicating that Koreans consumed larger quantities of various kinds of beverages when eating outside the home than eating at home [32]. According to the results of existing studies, adequate water intake and healthy beverage consumption should be particularly emphasized for those who have more opportunities to snack and eat outside the home.

The present study has both limitations and strengths. Firstly, it used cross-sectional data which has limitations in identifying causal relationships between water intake and energy and dietary intakes and eating behaviors. Secondly, we adjusted for sociodemographic and health-related variables as covariates. However, we did not consider the influence of seasonal variations in water intake [49] since the KCDC does not publicly release seasonal data collected from the KNAHENS. Therefore, future research considering the seasonal effect on water intake needs to be conducted when seasonal information is available. Despite these limitations, this study is significant in that it provides the first snapshot of water intake and beverage consumption in the Korean population using large-scale data. Finally, as mentioned earlier, the necessity of nationally representative data on water intake of Koreans should be emphasized. In the US, the US Department of Agriculture and the US Department of Health and Human Services have put considerable effort into understanding all forms of water intake in the US population for a long time [37]. In line with this, since 2005–2006, the NHANES has changed the method of collection of plain water intake data from the FFQ-type question to 24-h dietary recall methods [37,50,51]. This change has allowed for more accurate estimates of total water intakes in the US. However, this is not the case with the KNHANES. While intakes of water from beverages and foods have been estimated by 24-h dietary recalls, plain water intake has continued to be measured by the FFQ-type question. Thus, total water intakes from foods and beverages quantified in our study may not include plain water intake. It was also impossible to report the association of usual plain water intake obtained from the FFQ-type question with energy, macronutrients, and other dietary constituents reported by the 24-h dietary recall. Additionally, in general, a 24-h dietary recall method is preferred as a method to obtain more valid and accurate intake data than an FFQ [52]. Therefore, additional information on plain water intake, including tap and bottled water, should be collected by the 24-h dietary recall in the KNHANES, and further study using these data needs to be conducted.

## 5. Conclusions

In conclusion, our study quantified total water intake and investigated the important nutritional and diet-related behavioral contributors of water intake among Korean adults. Water intake from beverages and foods was positively associated with total daily energy intake, but negatively associated with the energy density of non-beverage foods. The present study also found strong and independent associations between water intake and eating location (eating outside the home vs. at home), types of meals (main meals and snacks), and eating episodes. This additional information might help to provide more efficient strategies for nutritional education to prompt adequate water intake among people with various characteristics of dietary intakes and meal consumption behavior. Future studies using plain water intake measured by 24-h dietary recalls rather than FFQs or simple questionnaires are needed in order to answer the question “What are the best water intake guidelines for Koreans to ensure the proper role of water in the body?”

## Figures and Tables

**Table 1:** Water intake by sociodemographic and health-related behaviors in Korean adults, KNHANES 2008–2012 ^1^.

Independent Variable	*n*	Water Intake from Beverages ^2^	Water Intake from Foods	Total Water Intake Excluding Plain Water	Usual Plain Water Intake
		g	g	g	mL
All, unadjusted	25,122	396 ± 4 ^3^	697 ± 4	1093 ± 6	1321 ± 65
All, adjusted for all covariates	25,122	398 ± 5	673 ± 5	1071 ± 7	1281 ± 67
Sex					
Men	10,184	426 ± 6	736 ± 6	1162 ± 10	1303 ± 61
Women	14,938	370 ± 8	610 ± 7	980 ± 11	1259 ± 108
*p* Value ^4^		<0.0001 **	<0.0001 **	<0.0001 **	0.6972
Age (years)					
19–29	2811	456 ± 12	592 ± 9	1048 ± 16	1564 ± 177
30–49	9248	445 ± 7	717 ± 8	1162 ± 11	1245 ± 91
50–64	7086	395 ± 7	762 ± 7	1157 ± 11	1218 ± 86
65+	5977	296 ± 6	621 ± 8	917 ± 11	1098 ± 144
*p* Value		<0.0001 **	<0.0001 **	<0.0001 **	0.1587
Income					
Q1 (lowest)	6092	379 ± 8	623 ± 7	1002 ± 11	1305 ± 146
Q2	6333	399 ± 7	665 ± 7	1064 ± 10	1408 ± 152
Q3	6354	400 ± 8	686 ± 7	1086 ± 11	1182 ± 85
Q4 (highest)	6343	414 ± 8	718 ± 9	1132 ± 12	1229 ± 100
*p* Value		0.0053 **	<0.0001 **	<0.0001 **	0.5995
Education level					
≤Elementary school	7014	337 ± 8	595 ± 9	932 ± 12	1415 ± 169
Middle school	2912	384 ± 10	668 ± 10	1052 ± 14	1220 ± 104
High school	8471	414 ± 7	702 ± 7	1116 ± 10	1398 ± 118
≥College	6725	456 ± 8	727 ± 7	1184 ± 11	1091 ± 76
*p* Value		<0.0001 **	<0.0001 **	<0.0001 **	0.0649
Smoking					
Never	15,302	323 ± 6	691 ± 6	1014 ± 9	1176 ± 77
Former	2863	438 ± 11	699 ± 11	1137 ± 16	1175 ± 133
Current	6957	433 ± 8	628 ± 7	1062 ± 11	1492 ± 109
*p* Value		<0.0001 **	<0.0001 **	<0.0001 **	0.0260 *
Day of recalled intake					
Monday-Thursday	15,503	398 ± 6	667 ± 6	1053 ± 9	1338 ± 81
Friday-Saturday	9619	398 ± 6	678 ± 6	1089 ± 10	1224 ± 99
*p* Value		0.0011 **	0.1498	0.0010 **	0.3454
Regular physical activity ^5^					
Yes	12,923	385 ± 6	685 ± 6	1083 ± 9	1342 ± 95
No	12,199	411 ± 7	660 ± 6	1059 ± 9	1220 ± 92
*p* Value		0.9370	<0.0001 **	0.0096 **	0.3487
Body mass index (kg/m^2^)					
<23	11,227	388 ± 6	656 ± 6	1044 ± 8	1324 ± 118
23 to <25	5957	401 ± 8	682 ± 8	1084 ± 11	1131 ± 88
≥25	7938	405 ± 7	680 ± 7	1085 ± 10	1388 ± 96
*p* Value		0.0426 *	0.0006 **	0.0001 **	0.0514

^1^ Data were from the Korea National Health and Nutrition Examination Surveys (KNHANES). All data except for sample size were weighted accounting for the complex study design according to the directions of the KNHANES analytical guidelines. All covariates shown in the table were added to regression models for all water variables; ^2^ Water intake from beverages (excluding plain water) and foods were estimated based on a 24-h dietary recall in the KNHANES 2008–2012. Total water intake excluding plain water was a combination of water intake from beverages and foods. Usual plain water intake were estimated from FFQ-type question after collection of the 24-h dietary recalls; ^3^ All values represented adjusted means ± standard errors (SEs); ^4^ *p* Value obtained from the multiple linear regression analyses indicated the significance of the association of each independent variable with all water variables (* *p* < 0.05, ** *p* < 0.01); ^5^ Having regular physical activity was defined as walking ≥5 time a week for ≥30 min each time.

**Table 2:** Water and energy intakes from beverages among Korean adults, KNHANES 2008–2012 ^1^.

Beverage Category	Consumers	Amount of Beverages Consumed		Water Intake from Beverages		% total Daily Energy Intake		% of Total Water Intake Excluding Plain Water	
		per Capita	per Consumer	per Capita	per Consumer	per Capita	per Consumer	per Capita	per Consumer
	*n* (Weighted %)	mL	mL	g	g	*%*	*%*	*%*	*%*
Milk	6170 (25.7) ^2^	73.5 ± 1.7	276.7 ± 3.8	62.1 ± 1.4	236.1 ± 3.3	2.3 ± 0.1	8.8 ± 0.1	5.6 ± 0.1	21.4 ± 0.2
Unsweetened coffee/tea	7745 (31.9)	108.0 ± 2.2	292.3 ± 4.8	100.2 ± 2.1	272.0 ± 4.5	0.3 ± 0.0	0.9 ± 0.0	8.3 ± 0.2	22.3 ± 0.3
100% fruit juice	1213 (5.2)	13.1 ± 0.6	247.4 ± 7.2	11.7 ± 0.6	222.5 ± 6.6	0.3 ± 0.0	5.4 ± 0.2	1.0 ± 0.0	18.3 ± 0.4
SSBs	11,644 (48.5)	139.7 ± 2.2	287.3 ± 3.2	122.5 ± 1.9	251.9 ± 2.8	3.0 ± 0.0	6.3 ± 0.1	11.2 ± 0.1	23.1 ± 0.2
Soft drink	9354 (37.2)	88.8 ± 1.5	238.1 ± 2.6	78.0 ± 1.3	209.2 ± 2.3	2.0 ± 0.0	5.4 ± 0.1	7.6 ± 0.1	20.5 ± 0.2
Fruit drink	1694 (7.7)	19.9 ± 0.8	251.6 ± 5.7	16.6 ± 0.7	211.6 ± 4.6	0.4 ± 0.0	5.5 ± 0.1	1.4 ± 0.1	18.4 ± 0.4
Sweetened coffee/tea	1771 (9.3)	26.6 ± 1.0	284.9 ± 6.0	23.8 ± 0.9	254.9 ± 5.5	0.5 ± 0.0	5.8 ± 0.1	1.9 ± 0.1	20.1 ± 0.4
Sports/energy drink	165 (0.9)	3.1 ± 0.4	333.7 ± 16.0	2.9 ± 0.3	312.1 ± 15.0	0.0 ± 0.0	4.0 ± 0.2	0.2 ± 0.0	21.9 ± 1.0
Other SSBs	223 (1.0)	1.3 ± 0.1	138.6 ± 6.8	1.2 ± 0.1	121.5 ± 6.0	0.0 ± 0.0	3.3 ± 0.2	0.1 ± 0.0	10.5 ± 0.5
Alcoholic beverages	4630 (20.9)	116.4 ± 2.9	557.2 ± 9.1	99.2 ± 2.5	474.6 ± 8.0	3.6 ± 0.1	17.1 ± 0.2	6.2 ± 0.1	29.7 ± 0.4
Diet beverages	16 (0.1)	0.2 ± 0.1	246.4 ± 30.6	0.2 ± 0.1	241.9 ± 27.9	-	0.2 ± 0.1	-	23.5 ± 3.0
Total beverages	19,900 (82.0)	451.0 ± 4.5	541.5 ± 4.7	395.8 ± 4.0	475.5 ± 4.1	9.6 ± 0.1	11.6 ± 0.1	32.4 ± 0.2	38.7 ± 0.2

^1^ Data were from the Korea National Health and Nutrition Examination Surveys (KNHANES). All data except for sample size were weighted accounting for the complex study design according to the directions of the KNHANES analytical guidelines. All values represented adjusted means ± standard errors (SEs), unless otherwise indicated. SSBs, sugar-sweetened beverages; ^2^ Values represented frequency (weighted percentage).

**Table 3:** Water intakes from beverages and foods associated with energy and nutrients intakes in Korean adults, KNHANES 2008–2012 ^1^.

Independent Variable	Water Intake from Beverages ^2^	Water Intake from Foods	Total Water Intake Excluding Plain Water
	g	g	g
Energy intake (100 kcal/day)	17.8 ± 0.5 ^3,4,^**	30.7 ± 0.5 **	48.5 ± 0.6 **
Energy from fat (5%)	21.9 ± 2.2 **	15.6 ± 2.2 **	37.5 ± 3.2 **
Energy from protein (5%)	−11.8 ± 4.2 **	120.2 ± 4.5 **	108.4 ± 6.4 **
Energy from carbohydrate (5%)	−54.1 ± 1.5 **	−4.6±1.6	−59.0 ± 2.1 **
Energy density of all beverages (kcal/g)	−43.9 ± 2.9 **	−1.0 ± 2.8 **	−44.9 ± 3.9 **
Energy density of all non-beverage foods (kcal/g)	−0.8 ± 7.4 **	−530.3 ± 7.2 **	−531.1 ± 9.9 **
Fiber (5 g/day) ^5^	−57.4 ± 4.7 **	173.9 ± 5.3 **	116.5 ± 4.4 **
Sodium (100 mg/day) ^5^	−1.7 ± 0.1 **	3.4 ± 0.1 **	1.7 ± 0.2 **
Calcium (100 mg/day) ^5^	7.1 ± 1.4 **	25.8 ± 1.9 **	32.9 ± 2.5 **
Amount of beverages consumed (100 g/day)	85.0 ± 0.3 **	2.9 ± 0.7 **	87.9 ± 0.8 **
Amount of foods consumed (100 g/day)	2.5 ± 0.6 **	64.1 ± 0.8 **	66.6 ± 1.1 **
Usual plain water intake (100 g/day)	0.0 ± 0.0	0.1 ± 0.1	0.0 ± 0.1
Water in foods (100 g/day)	3.0 ± 0.8 **	-	103.0 ± 0.8 **
Water in beverages (100 g/day)	-	3.1 ± 0.8 **	103.1 ± 0.8 **
Water in milk (100 g/day)	82.4 ± 2.2 **	0.1 ± 2.8	82.5 ± 3.5 **
Water in unsweetened coffee/tea (100 g/day)	83.5 ± 1.3 **	−0.8 ± 1.5	82.7 ± 1.9 **
Water in 100% fruit juice (100 g/day)	87.5 ± 4.4 **	14.1 ± 4.5 **	101.6 ± 6.5 **
Water in SSBs (100 g/day)	78.5 ± 1.5 **	2.5 ± 1.8	81.0 ± 2.4 **
Water in alcoholic beverages (100 g/day)	90.9 ± 0.8 **	4.7 ± 1.1 **	95.5 ± 1.4 **
Water in diet beverages (100 g/day)	32.5 ± 21.5 **	−50.3 ± 27.0	−17.9 ± 31.0

^1^ Data were from the Korea National Health and Nutrition Examination Surveys (KNHANES). All data except for sample size were weighted accounting for the complex study design according to the directions of the KNHANES analytical guidelines. Water intake from beverages (excluding plain water) and foods were estimated based on a 24-h dietary recall in the KNHANES 2008–2012. The multiple regression models included covariates including sex, age (continuous), income (low, mid-low, mid-high, or high) and education levels (elementary school graduates, middle school graduates, high school graduates, or more than college graduate), smoking status (non-smoker, former smoker, or current smoker), the day of recalled intake (Monday-Thursday or Friday-Sunday), regular physical activity (yes or no), and body mass index (continuous). SSBs, sugar-sweetened beverages; ^2^ Water intake from beverages (excluding plain water) and foods were estimated based on a 24-h dietary recall in the KNHANES 2008–2012. Total water intake excluding plain water was a combination of water intake from beverages and foods; ^3^ All values represented βs ± standard errors (SEs) which were associated with units of measurement given in parentheses for each independent variable (for example, when there was a 100 kcal/day increase in energy intake, water intake from beverage increased by 18 g, water intake from food increased by 31 g, and total water intake excluding plain water increased by 49 g); ^4^ *p* Values obtained from the multiple linear regression analyses indicate the significance of the association of each independent variable with all water variables (* *p* < 0.05, ** *p* < 0.01); ^5^ The models also included total daily energy (continuous) intake as an independent variable.

**Table 4:** Water intakes from beverages and foods associated with eating behaviors in Korean adults, KNHANES 2008–2012 ^1^.

Independent Variable	Water Intake from Beverages ^2^	Water Intake from Foods	Total Water Intake Excluding Plain Water
	g	g	g
Number of different beverage items consumed ^3^	211.2 ± 2.7 ^4,5,^**	−38.5 ± 3.0 **	172.6 ± 3.8 **
Number of different non-beverage food items consumed ^3^	−0.01 ± 1.0	14.6 ± 0.9 **	14.6 ± 1.2 **
Number of all eating episodes ^3^	62.1 ± 2.1 **	9.0 ± 1.8 **	71.1 ± 2.3 **
Number of all snack episodes ^3^	71.2 ± 2.0 **	6.0 ± 1.8 **	77.3 ± 2.3 **
Number of beverage-only episodes ^3^	112.3 ± 2.4 **	−18.8 ± 2.2 **	93.5 ± 2.9 **
Length of ingestion period (h) ^3^	14.1 ± 1.2 **	0.4 ± 1.0	14.6 ± 1.5 **
Reported breakfast in the 24-h dietary recall ^3^	−103.8 ± 8.9 **	40.0 ± 7.3 **	−63.8 ± 10.0 **
Reported any snack in the 24-h dietary recall ^3^	158.7 ± 7.1 **	45.0 ± 6.9 **	203.7 ± 9.4 **
Energy from main meals (5%)	−4.7 ± 0.4 **	5.6 ± 0.4 **	0.9 ± 0.6
Energy from snacks (5%)	30.2 ± 1.2 **	11.9 ± 1.1 **	42.1 ± 1.7 **
Energy from eating outside home (5%)	12.6 ± 0.6 **	2.1 ± 0.5 **	14.7 ± 0.8 **

^1^ Data were from the Korea National Health and Nutrition Examination Surveys (KNHANES). All data except for sample size were weighted accounting for the complex study design according to the directions of the KNHANES analytical guidelines. The multiple regression models included covariates including sex, age (continuous), income (low, mid-low, mid-high, or high) and education levels (elementary school graduates, middle school graduates, high school graduates, or more than college graduate), smoking status (non-smoker, former smoker, or current smoker), the day of recalled intake (Monday-Thursday or Friday-Sunday), regular physical activity (yes or no), and body mass index (continuous); ^2^ Water intake from beverages (excluding plain water) and foods were estimated based on a 24-h dietary recall in the KNHANES 2008–2012. Total water intake excluding plain water was a combination of water intake from beverages and foods; ^3^ The models also included total daily energy (continuous) intake as an independent variable; ^4^ All values represented βs ± standard errors (SEs) which were associated with units of measurement given in parentheses for each independent variable (for example, when the number of different kind of beverage items reported increased, water intake from beverages increased by 211 g, water intake from foods decreased by 39 g, and total water intake excluding plain water increased by 173 g); ^5^ *p* Values obtained from the multiple linear regression analyses indicate the significance of the association of each independent variable with all water variables (* *p* < 0.05, ** *p* < 0.01).

## Supplementary Materials

The following are available online at www.mdpi.com/2072-6643/8/10/617/s1, Table S1: Odds ratios and 95% confidence intervals of beverage consumption according to sociodemographic and health-related characteristics in Korean adults, KNHANES 2008–2012 ^1^.
