# Supplementary material for: Total Water Intake from Beverages and Foods Is Associated with Energy Intake and Eating Behaviors in Korean Adults
Source: Nutrients. 2016 Oct 4;8(10):617. doi: 10.3390/nu8100617 (PMC5084005; doi:10.3390/nu8100617)
Supplement: Supplementary file 1 [file nutrients-08-00617-s001.pdf]

Supplementary Materials: Total Water Intake from Beverages and Foods Is Associated with Energy Intake and Eating Behaviors in Korean Adults

Kyung Won Lee, Dayeon Shin and Won O. Song

Table S1. Odds ratios and 95% confidence intervals of beverage consumption according to sociodemographic and health-related characteristics in Korean adults, KNHANES 2008–2012 [1].

	Milk (*n* = 6170) [2]	Unsweetened Coffee/Tea (*n* = 7745)	100% Fruit Juice (*n* = 1213)	SSBs [3] (*n* = 11,644)	Alcoholic Beverages (*n* = 4630)
	AOR (95% CI)	*AOR (95% CI)*	*AOR (95% CI)*	*AOR (95% CI)*	*AOR (95% CI)*
Sex					
Men	1.00	1.00	1.00	1.00	1.00
Women	1.26 (1.13–1.41) [4,**]	1.39 (1.25–1.55) **	1.17 (0.93–1.46)	1.14 (1.04–1.25) **	0.83 (0.73–0.93) **
Age (years)					
19–29	1.00	1.00	1.00	1.00	1.00
30–49	0.75 (0.66–0.83) **	1.33 (1.18–1.49) **	0.82 (0.66–1.01)	1.27 (1.14–1.40) **	1.30 (1.12–1.50) **
50–64	0.79 (0.69–0.89) **	1.58 (1.38–1.80) **	0.83 (0.64–1.07)	0.96 (0.85–1.08)	1.44 (1.22–1.69) **
65+	0.77 (0.67–0.90) **	1.05 (0.90–1.22)	0.55 (0.40–0.76) **	0.96 (0.84–1.10)	1.06 (0.87–1.28)
Income					
Low	1.00	1.00	1.00	1.00	1.00
Mid-low	1.18 (1.05–1.32) **	1.06 (0.96–1.17)	0.98 (0.78–1.24)	1.01 (0.92–1.11)	0.96 (0.84–1.08)
Mid-high	1.13 (1.00–1.27) *	1.14 (1.02–1.27) *	0.98 (0.78–1.23)	0.99 (0.90–1.09)	1.06 (0.94–1.21)
High	1.11 (0.97–1.23)	1.23 (1.10–1.38) **	1.12 (0.90–1.40)	1.00 (0.91–1.11)	0.99 (0.87–1.13)

Education					
≤Elementary school	1.00	1.00	1.00	1.00	1.00
Middle school	1.26 (1.09–1.47) **	1.39 (1.23–1.58) **	1.10 (0.81–1.49)	1.14 (1.02–1.29) *	1.30 (1.11–1.53) **
High school	1.47 (1.29–1.68) **	1.66 (1.47–1.88) **	1.20 (0.92–1.55)	1.20 (1.07–1.34) **	1.15 (0.99–1.33)
≥College	1.87 (1.62–2.16) **	1.96 (1.71–2.24) **	1.40 (1.05–1.86) *	1.36 (1.21–1.53) **	1.01 (0.85–1.19)
Smoking					
Never	1.00	1.00	1.00	1.00	1.00
Former	1.18 (1.02–1.36) *	1.84 (1.60–2.11) **	1.05 (0.81–1.35)	1.16 (1.03–1.31) *	1.73 (1.49–2.02) **
Current	0.71 (0.63–0.79) **	1.08 (0.96–1.21)	0.80 (0.65–1.05)	1.18 (1.07–1.30) **	2.09 (1.85–2.37) **
Physical activity [5]					
Yes	1.00	1.00	1.00	1.00	1.00
No	0.88 (0.82–0.98) **	1.04 (0.97–1.12)	1.03 (0.89–1.19)	1.11 (1.04–1.18) **	1.06 (0.97–1.15)
Day of recalled intake					
Monday-Thursday	1.00	1.00	1.00	1.00	1.00
Friday-Saturday	1.02 (0.93–1.11)	1.17 (1.06–1.29) **	1.10 (0.94–1.28)	0.95 (0.88–1.02)	1.20 (1.09–1.31) **
Body mass index (kg/m^2)					
<23	1.00	1.00	1.00	1.00	1.00
23 to <25	0.96 (0.87–1.05)	1.02 (0.94–1.11)	1.01 (0.84–1.23)	0.99 (0.91–1.07)	1.05 (0.95–1.16)
≥25	0.90 (0.82–0.98) *	1.14 (1.05–1.23) **	0.98 (0.82–1.17)	0.93 (0.86–1.00)	1.00 (0.90–1.10)

[1] Data were from the Korea National Health and Nutrition Examination Surveys (KNHANES). All data except for sample size were weighted accounting for the complex study design according to the directions of the KNHANES analytical guidelines. The multiple logistic models included sex, age (continuous), income (low, mid-low, mid-high, or high) and education levels (elementary school graduates, middle school graduates, high school graduates, or more than college graduate), smoking status (nonsmoker, former smoker, or current smoker), the day of recalled intake (Monday-Thursday or Friday-Sunday), physical activity (yes or no), body mass index (continuous), and total daily energy intake (continuous). Diet beverage category was excluded from the multiple logistic regression due to the small sample size of consumer (n = 16). SSBs, sugar-sweetened beverages; AOR, adjusted odds ratio; 95% CI, 95% confidence interval; [2] n referred to consumers of each beverage group; [3] SSBs included sodas, fruit drinks, sweetened coffees/teas, sports/energy drinks, and other SSBs; [4] Significant associations were indicated in bold (* $p < 0.05$, ** $p < 0.01$); [5] Having physical activity was defined as walking ≥5 time a week for ≥30 min each time.
